# Interactional convergence in conversational storytelling: when reported speech is a cue of alignment and/or affiliation

**DOI:** 10.3389/fpsyg.2013.00705

**Published:** 2013-10-08

**Authors:** Mathilde Guardiola, Roxane Bertrand

**Affiliations:** Laboratoire Parole et Langage UMR 7309, CNRS, Aix Marseille UniversitéAix-en-Provence, France

**Keywords:** conversation, convergence, alignment, affiliation, similarity, storytelling, reported speech, French

## Abstract

This paper investigates how and when interactional convergence is established by participants in conversation. We analyze sequences of storytelling using an original method that combines *Conversation Analysis* and a *corpus-based* approach. In storytelling, the participant in the position of “listener” is expected to produce either generic or specific responses adapted to the storyteller's narrative. The listener's behavior produced within the current activity is a cue of his/her *interactional alignment*. We show here that the listener can produce a specific type of (aligned) response, which we term a *reported speech* utterance *in echo*. The participant who is not telling the story is nonetheless able to animate the characters, while reversing the usual asymmetric roles of storyteller and listener. The use of this device is a way for the listener to display his/her stance toward the events told by the storyteller. If the listener's stance is congruent with that of the storyteller, this reveals a high degree of *affiliation* between the participants. We present seventeen excerpts from a collection of 94 instances of Echo Reported Speech (ERS) which we examined using the concepts of alignment and affiliation in order to show how different kinds of convergent sequences are constructed. We demonstrate that this phenomenon is mainly used by the listener to align and affiliate with the storyteller by means of reformulative, enumerative, or overbidding ERS. We also show that in affiliative sequences, reported speech can be used by the listener in a humorous way in order to temporarily disalign. This disalignment constitutes a potential starting point for an oblique sequence, which, if accepted and continued by the storyteller, gives rise to a highly convergent sequence.

## Introduction

The goal of this paper is to describe the resources and means used by participants to create convergent sequences in face-to-face interactions in French. Since Sacks and colleagues published the first papers (see Sacks et al., 1974, among others) in the field of *Conversational Analysis*, it has been demonstrated that a face-to-face interaction is a collaborative production by all the participants. For example, while the main speaker is talking, the listener plays an active role through short utterances or backchannel signals to show sustained attention and understanding of the discourse (Schegloff, 1982). Simultaneously, backchannel signals provide information about the processes the speaker uses to mark important steps in the discourse (Fox Tree, 1999). Although this collaborative production or *joint-construction* is a necessary requirement for successful interaction (Clark, 1996), it does not necessarily produce convergent sequences. Here we describe the specific points that allow these sequences to emerge.

We rely on the notions of *alignment* and *affiliation*, as defined in *Conversational Analysis* by Stivers (2008), to investigate when and how convergent sequences can appear in conversation. Drawing on conversational storytelling sequences, we focus on reported speech, i.e., speech or thoughts attributed to another person and another context (Holt, 1996; Bolden, 2004). We attempt to demonstrate that for the listener who uses it, this discursive device, usually expressed by the main speaker, constitutes a good candidate for alignment and affiliation.

The *Conversational Analysis* framework (henceforth *CA*) aims to describe social activities in which speakers attempt to accomplish goals in interaction. Among the various approaches within CA, we adopt the *Interactional Linguistics (IL)* approach (Couper-Kuhlen and Selting, 1996; Barth-Weingarten et al., 2010, among others). This approach provides a systematic method for studying how and what kind of resources (prosodic, syntactic, semantic, gestural, and so on) participants deploy to manage talk-in-interaction. From an *IL* perspective, the purpose of an interaction is to accomplish actions (questions, repairs) and activities such as story-telling, arguing, disputing, describing, and direction-giving (Selting, 2010). Interactions are heterogeneous; they generally include more than one activity, and participants take on different recognizable discursive roles during different activities that impact the organization of turn-taking (Szczepek Reed, 2010). We chose to focus on convergence within one activity in particular: *storytelling*.

Storytelling very frequently occurs in conversation and has been investigated not only to improve the definition of the conversational unit but also to characterize participants' roles and turn-management (Selting, 2000). In storytelling, the main speaker needs several *Turn-Constructional Units* (*TCUs*) to reach the end of his/her story and to make the transition to another speaker possible [a *Transition Relevance Place (TRP)*]. Because the storyteller (the main speaker) and the listener have different storytelling roles, storytelling is seen as an asymmetrical activity, but both participants actively participate and work together to construct the narrative.

The main speaker has to respect the expectations that participants have of storytelling. First of all, the speaker has to ensure that he/she can begin to tell the story. A story is a *large project* (Selting, 2000) implying a long duration, so the main speaker has to be authorized by the listener to tell it. The story itself has to be “tellable”: it should present an interest. Moreover, the story has to be told in a specific order that has been described as a succession of formal phases (Labov and Waletzky, 1966). In the first two phases, the *orientation* and *complication* phases, the storyteller presents characters and events, respectively. The *apex* corresponds to the culminating point of the story, after which a kind of evaluation phase can appear. Stories in conversation can also exhibit other phases such as *parenthesis* or *aside* (Selting, 2000).

The listener also has to comply with certain expectations. He/she is supposed to listen to the story while providing feedback showing ratification of the storyteller in this role, understanding of the ongoing discourse, and the state of shared knowledge, for instance. Bavelas et al. (2000) have shown that the responses produced by the listener in storytelling are so important that the teller cannot tell the story correctly when responses are absent or perturbed. By respecting these obligations, the listener achieves alignment during the activity. *Alignment* is defined “*with respect to the activity in progress”* (Stivers, 2008: 34). For our study, a type of aligned behavior by the listener would be to produce responses matching the speaker's expectations. At the beginning of the story, the listener begins to align him/herself as a story recipient using appropriate responses (Jefferson, 1978), for example. More generally, Bavelas et al. (2000) have described listeners' responses as *generic* or *specific*: the first simply correspond to responses required by the activity that are sufficiently general to adapt to any type of narrative, while the second are specifically adapted to the ongoing narrative. The authors have shown that specific responses appear later in the narrative than generic ones. In line with these results, a previous study on our French conversational corpus (Guardiola et al., 2012) showed that morpho-syntactically richer responses labeled as specific responses mainly appeared later in the narration. These results suggest that generic responses produced earlier than specific responses require less knowledge of the situation described by the storyteller. In our opinion, generic responses function as *continuers* or *acknowledgements* (Schegloff, 1982), which help to show how shared knowledge is elaborated during the initial phases of stories, while specific responses function as *assessments* (evaluative or attitudinal) once sufficient information about the story has been provided.

The information given by the teller includes his/her own stance on the events, and the stance the listener is expected to have. Giving *affiliative* responses, for the listener, thus, means providing the expected stance toward the story. *Affiliation* is defined as the fact that *“the hearer displays support of and endorses the teller's conveyed stance”* (Stivers, 2008: 35). Stivers develops the argument that affiliation requires alignment. Since affiliation implies that the participant knows the teller's stance on his/her own story (and knows that a similar stance is the preferred response), it means that the participant has gathered enough information about the story being told, which is shown by displaying alignment.

In this paper, we are interested in exploring the emergence of convergent sequences in terms of the concepts of alignment and affiliation. In any domain, convergence is usually defined as a behavior that becomes more and more similar over the time. For us, a convergent sequence requires preliminary alignment and affiliation, associated with similarity (including at the phonetic, prosodic, syntactic, semantic, lexical, and/or discursive levels). We consider a sequence as convergent when the interactional statuses evolve toward symmetry between participants.

We argue that a single alignment is not sufficient to produce convergent sequences; they also require affiliation. To achieve affiliation, in turn, participants need to share sufficient common ground, which they co-elaborate during the first part of the story: the main speaker gives new information while the listener shows, through generic responses for example, that he/she adds them to his/her own common ground (alignment).

Direct reported speech is a very frequent discursive device used by the storyteller around the apex of the narrative (Holt, 2000; Blondal, 2005). Most of the time, the storyteller, while apparently reporting speech in an objective way, gives many implicit cues of his/her own stance toward the events. The listener then understands the teller's stance and is thus, able to explicitly produce the same stance as the teller, which constitutes the *preferred response*. This creates a highly affiliative sequence in interaction (Holt, 2000). Storytellers use reported speech in order to elicit affiliation from the listener, and listeners' reactions to reported speech show their affiliation. Reported speech has been described in the literature as a device exhibited by the main speaker for reporting words that have already been uttered in another situation. However, we demonstrate here that listeners themselves use direct reported speech to show their affiliation. The data show that some of the specific responses produced by the listener take the form of *direct reported speech utterances produced by the listener*, henceforth, “Echo Reported Speech” (ERS). In the cases we study here, the participant who produces the reported speech is not the teller, so he cannot have heard these words before. They thus, have an “inventive” function (see Vincent and Dubois' typology, 1997). But more importantly, by using this device, the listener reveals that the canonical roles played by the storyteller and the listener can be temporarily reversed.

We use a sequential analysis to explore how reported speech offers a way for the listener to exhibit a form of (dis)alignment and (dis)affiliation. In the sequential analysis, an utterance is considered in relation to the preceding and the following turns. The action accomplished by a turn is revealed by the context in which it occurs, but also by the action's consequences on the *interactional orientation* of the sequence (Hutchby and Wooffitt, 1998). A turn by the listener may or may not be ratified by the storyteller, and the storyteller can then orient either toward the listener's turn or toward his/her own previous turns (storytelling), while ignoring the listener's turn.

By analyzing our instances of ERS using the concepts of alignment and affiliation, we provide evidence of the emergence of highly convergent sequences. Instances of ERS are examined in order to study their link with both alignment and affiliation. Stivers (2008) claims that alignment and affiliation are the result of different phenomena, showing for instance that *nodding* reveals affiliation while a vocal *mh* reveals alignment. However, we believe that any device can be used for one or both of these dimensions. We thus, demonstrate that the use of the same device—ERS—can result in dissimilarly convergent sequences (local or large-span convergent sequences, or sometimes non-convergent sequences).

In the section Materials and Methods, we present the corpus and the method used. After some descriptive data, the main focus of the section Results and Analysis is the sequential analysis of several examples, which allows us to argue for an interactional description of ERS in terms of alignment and affiliation. In section Discussion, we discuss the implications of these notions on the potential emergence of a convergent sequence, and we present perspectives for further work on interactional convergence.

## Materials and methods

### Corpus

**Figure 1:**
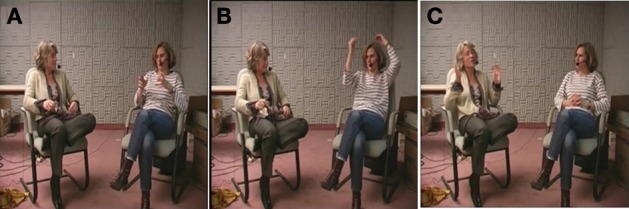
**Experimental setup for the *CID***.

The study was lead on the *Corpus of Interactional Data (CID)* (Bertrand et al., 2008). Figure 1 shows the experimental setup: the corpus (i.e., 8 one-hour French dialogues) was recorded in an anechoic room; each speaker is wearing a microphone; and the positions and proximity of the participants indicates that they are having a conversation.

The participants were given instructions to tell personal stories: unusual stories for half of them, and stories about work conflicts for the other half. This kind of task (telling amusing stories and making complaints) is known to promote the occurrence of reported speech (Holt, 2000). The instructions might have led the participants to engage in an asymmetrical interaction, but this was not the case. The data closely resemble what we consider a natural conversation (Bertrand et al., 2008): the participants did not have a third party to manage turn-taking and were free to negotiate their roles as listeners or tellers. In addition, they were familiar with the place where they were being recorded and knew each other well, allowing them to adopt a very informal style during the interactions.

### Methodological approach

Our method combines *IL* and a *corpus-based approach* (corpus processing). We adopted this dual approach in order to systematically annotate the phenomena in the corpus. We then drew up a list of occurrences of similar events in our dataset, i.e., a collection of examples, as recommended in *CA* (Mondada, 2013). However, unlike a *CA*-style transcription that exhibits all the relevant cues (prosodic, gestural, phonetic) on the same line (see (Selting et al., 1998) for the *GAT* system, adapted from the Jefferson-style transcription system), the transcription used in this work is one of the levels of the annotation process elaborated within the framework of the *OTIM* project (Blache et al., 2009). Using Praat (Boersma and Weenink, 2009), rich and systematic annotations related to the different linguistic domains, from phonetic to gestural, are provided. For each domain, the link between the annotated phenomena is encoded using an annotation scheme that requires a certain degree of formalization (see below for the annotation scheme used for reported speech). Precise synchronization between the different annotations makes it possible to study the relationships between them.

For the present study, we used the following annotation levels: orthographic transcription, tokens, prosodic units (phrasing) and pitch contours, narratives, morpho-syntactic categories, speech overlap phases and laughter. For each speaker, all of the information is aligned in time with a precision level of one phoneme. This gave us a very precise description of the timing and delay of any phenomenon in the corpus. Considering this, we can study the co-occurrence of several phenomena.

### The annotation scheme used for direct reported speech

**Figure 2:**
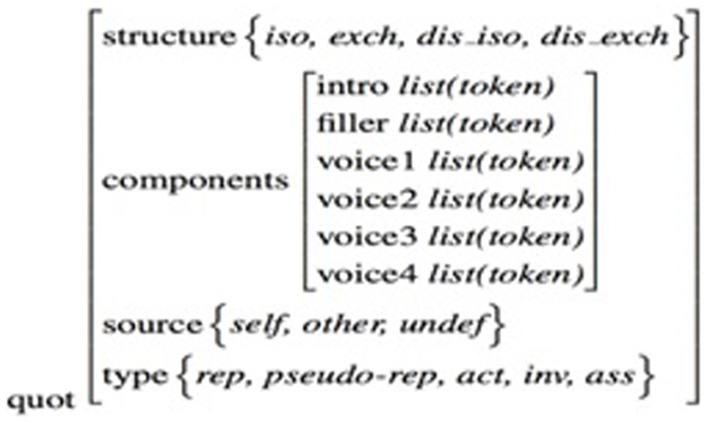
**Annotation scheme of direct reported speech in the CID**.

As this study focuses on reported speech, we only present, in Figure 2, the relevant parts of the overall annotation scheme (Blache et al., 2010). The reported speech sequences were annotated along several dimensions:

– *Q*(uotation)-*structure:* the (optional) exchange structure, which reveals whether the scene reports speech from one character or more than one character (in a reported dialogue).
– *Q-component*: the various components of the structure (introductive formula, the different voices, etc.).
– *Q-source:* the origin of the voices used by the speaker.
– *Q-type*: the functional type of quotation based on the typology of Vincent and Dubois (1997): *Reproduction* consists of presenting the reported speech as having already been said in another situation. *Pseudo-reproduction* corresponds to reported speech for which the previous situation is not clearly identifiable. *Assertion* is the fact of reporting speech as an authority argument. *Actualization* is used to report speech that has occurred in several similar situations. In *invention*, the speaker presents the speech as never having been said.

Reported speech utterances were first located by a transcriber and then annotated by two other experts according to the annotation scheme presented above. The corpus-based method implies several annotators between whom a score of agreement is calculated. For our study, we retained only the cases where the two experts agreed. Reported speech was annotated for each speaker over the course of the whole interaction, regardless of the discursive role of the participant at each point of the interaction.

Once the reported speech was annotated, we conducted a manual *turn-by-turn analysis* of the sequences (*CA* approach). To analyze a turn using this type of analysis, we determined which actions the previous turn achieved and took into account the next turn, which gives cues about how the target turn is received by the participants. This last point raises the question of ratification: to consider that an action has been achieved in interaction, one should carefully observe its possible consequences on the interaction, especially its ratification by the interacting participants.

The results discussed below show how the participants aligned and affiliated using ERS to co-construct convergent sequences in conversation.

## Results and analysis

### Descriptive data

Let us consider the results of eight dialogues we selected for analysis. Table 1 below shows the number of narratives and the speech production time per speaker, as well as the distribution of reported speech between the storyteller and the listener (ERS).

**Table 1:** **Descriptive data for each speaker: narratives, speech time, reported speech and ERS instances**.

**Dialogue**	**Speaker**	**Narrative occurrences**	**Speech time (in s)**		**Q_structure utterances**	
			**As narrator**	**As listener**	**Non-echo**	**ERS instances**
AB-CM	AB	7	911.4	108.9	45	10
	CM	6	639.5	212.1	20	8
AC-MB	AC	8	634.4	180.0	33	10
	MB	17	1304.1	61.1	51	3
AG-YM	AG	14	606.5	71.8	30	2
	YM	13	485.7	101.1	47	1
AP-LJ	AP	10	422.4	94.0	34	13
	LJ	8	389.9	73.3	46	13
BX-MG	BX	6	282.1	16.7	11	2
	MG	5	202.7	10.7	18	1
EB-SR	EB	9	403.5	62.6	18	17
	SR	10	681.1	41.2	37	7
IM-ML	IM	13	730.6	105.8	30	2
	ML	12	631.3	74.44	40	5
LL-NH	LL	2	119.1	64.92	9	0
	NH	7	546.8	8.2	27	0
Total		147			496	94

Out of the 590 quotation structures annotated, we found 94 occurrences of ERS. Every participant (except for the LL-NH pair) used ERS at least once during the interaction. Eight listeners produced at least five reported speech utterances, and in three dialogues each listener used about 15 reported speech utterances, showing how frequently ERS appears in storytelling. Three of the dialogues show an asymmetric ERS distribution, with more occurrences produced by one of the participants (17 vs. 7 for EB-SR, 10 vs. 3 for AC-MB, 5 vs. 2 for IM-ML). One of the listeners (AC) who used more reported speech produced fewer narrative sequences. This listener seemed to adopt the listener role more frequently throughout the dialogue. Lastly, the dialogue where ERS was absent (LL-NH) is atypical in the sense that it contains far fewer narratives than the other dialogues (2 and 7 narratives), and one of the participants (LL) did not speak much.

### Description of echo reported speech

As shown in our data, ERS is the listener's invention of what reported speech would sound like at an event where the listener was not present. This type of reported speech was found in the middle or at the end of the narrative (around the apex or evaluation phases), as expected. It was produced in the very close environment of reported speech, either during an occurrence or a sequence of canonical direct reported speech produced by the storyteller.

Let us now examine a rather typical case of ERS (Ex1). This extract occurs at the apex of the story, where MB first produces reported speech (in her own voice) to ask a child a question (line 630d). The listener, AC, produces specific responses (lines 991, 992), before producing reported speech with the fictitious voice of the child and answering MB's question (line 993). While doing this, AC creates a fictitious dialogue between MB (herself) and the child. MB then continues her activity of reporting this exchange between them. The use of deixis (line 993) is coherent for storytelling, not for the current recording situation (the dialogue *hic et nunc*): AC is not speaking with her own voice, so she must be reporting speech.

Ex1_Fr:


   *MB_630d voilà et donc je vois le gamin avec
        le bras dans le plâtre il me dit
        j suis tombé de l'escabeau je dis
        qu'est-ce tu fais sur un escabeau
        tu vois je comprenais pas trop
  AC_991 putain
  AC_992 ça craque
→ **AC_993 je v- je vais sur le balcon de la
         voisine @**
 MB_631d je croyais pour attraper des trucs
         au mur il me dit c'est sur le
         balcon je dis
 MB_632d un escabeau sur le balcon mais tu
         es fou c'est hyper dangereux
  MB_633 il me dit non je le fais tous les
         soirs*


Ex1_En:


 MB_630d there + and so I see the kid with
         his arm in a cast he tells me I
         fell off the ladder I say what were
         you doing on a ladder you see I
         didn't understand him very well
  AC_991 shit
  AC_992 it cracks
**→ AC_993 I wen- @ I went out on the
         neighbor's balcony @**
 MB_631d I thought for getting some stuff
         down from the wall he tells me it's
         on the balcony I say
 MB_632d a ladder on the balcony but you're
         crazy that's dangerous
  MB_633 he tells me § no I do it every night


After some evaluative responses, turn 993 presents the characteristics of direct reported speech—change of verbal tense, deictics anchored in the reported situation- although it is produced by the participant who is in the position of receiving the story. This speaker has understood the described situation well enough to be able to continue the narrative, adding reported speech and making the characters talk, even though she had not witnessed the scene. This reported speech is thus, an invention, but it is anchored in the reported situation and specifically oriented toward the previous turn since it answers MB's previous question.

We now turn to the question of how ERS enables listeners to align and affiliate with the ongoing story and allows the conversation participants to initiate a convergent sequence.

### Sequential analysis

The listener mainly produced ERS just after a reported speech utterance produced by the storyteller. In doing so, the listener can either adopt the same voice the narrator was using, use another voice to build a reported dialogue, or even add a different tonality. In some cases, ERS anticipates the storyteller's reported speech production. In other cases, the use of reported speech initiates a new sequence, which will be considered separately.

The examples below (2–6) present the most common occurrences and are the most meaningful for showing affiliation.

#### Reformulation

The next two examples show cases of “similar voices” in reformulation.

In Example 2, AB is telling a story about a friend canceling a movie date. She reports a dialogue between her friend and herself. The ERS in 642 is a reformulation of the reported answer of AB, which is a commentary she made at the moment, in the situation being told. Although the ERS doesn't exhibit explicit cues of reported speech, it would be irrelevant in the present dialogue. It is indeed a reformulation, in a reported way, and expresses the same voice as AB (line 442). It is an aligned response in that it supports the ongoing story. At the same time, by doing this, CM displays the same stance as AB toward the event: canceling the date was not a serious problem. This reported speech utterance is thus, an affiliative response to the story told by AB. AB can consider this response as a backchannel (BC) signal. In line with Kern (2007), in some cases where a BC is expected, the lack of ratification is equivalent to a minimal ratification via a backchannel. So AB does not need to ratify it, in the sense that it is “normal” that CM's response is aligned and affiliative: if it were not, it would be signaled by a repair sequence. Therefore, in this case, the ERS is ratified by not saying anything.

Ex2_Fr:


  *AB_440 elle avait une gastro donc elle m'a
       dit
  CM_640 ah ouais
  AB_441 je suis malade depuis hier soir et
  CM_641 oui
  AB_442 je dis c'est pas grave
**→ *CM_642 tant pis***
  AB_443 c'est pas grave c'est pas grave on
         fera ciné à un autre moment*


Ex2_En:


  AB_440 she had a stomach ache so she told
       me
  CM_640 oh yeah
  AB_441 I've been sick since last night and
  CM_641 yes
  AB_442 I say it's no big deal
**→ CM_642 too bad**
  AB_443 no big deal no big deal we'll go to
         the movies another time


In some cases not only is the device similar, but so is the prosody and lexicon. In Example 3, IM is telling a story while criticizing the characters (teachers), who take breaks all day long. IM displays her stance by using a specific voice when she reports their (invented) speech (line 581). ML also animates the same figures (line 554) by using similar words and a similar prosodic delivery. The various reported speech occurrences (from both IM and ML) are produced in three intonative units: each is introduced with an open and lengthened “oh,” is ended with a rising pitch contour on the penultimate syllable and followed by a high plateau on the final schwa. This prosodic delivery sounds like a Southern French accent but its exaggerated production (Couper-Kuhlen, 1999; Günthner, 1999) contributes to exhibiting the criticism of the characters. By using the same delivery as IM (*prosodic stylization*, Szczepek Reed, 2006), ML displays the same stance and consequently shows affiliation.

Ex3_Fr:


  *IM_577 attention hein déjà on rentre un
         petit peu après l'heure dite
  ML_552 ouais ouais
  IM_578 à neuf heures
  IM_579 après on s'en re- on s'en refait
         une de au lieu d'une demi heure
         allez on fait trois quarts d'heure
  ML_553 ouais
  IM_580 l'après midi han
  IM_581 oh c'est déjà l'heure
**→ *ML_554 @ oh oh c'est déjà deux heures et
         quart oh vite il faut sonner la
         cloche @***
  IM_582 i- alors euh vite il faut rentrer
  IM_583 à quatre heures et quart ils les
         font rentrer et à quatre heure et
         demi ça sonne quoi*


Ex3_En:


  IM_577 hey listen already we're going back
         a little later than scheduled
  ML_552 yeah yeah
  IM_578 at nine o'clock
  IM_579 afterwards we'll take- we'll take
         another one of instead of a half
         hour come on let's do forty-five
         minutes
  ML_553 yeah
  IM_580 in the afternoon + uh
  IM_581 oh it's already time
**→ ML_554 @ whoa it's already two fifteen go
         quick we have to ring the bell @**
  IM_582 so uh quick we have to go back
  IM_583 at four-fifteen they make them go
         back and at four-thirty it rings,
         you know


*In sum*, for the two examples above (2 and 3), one can see that when the listener of a story animates a character, it constitutes an aligned and affiliative response for the ongoing narrative. Since these ERS utterances only reformulate what has just been reported by the storyteller, they do not require any explicit ratification by the storyteller.

#### Overbidding ers

The next example (Ex4) displays another type of affiliative ERS: overbidding ERS. AC is complaining about students' parents. She reports her own speech, which she virtually addresses to the parents. MB also produces reported speech using the same voice as AC, showing that she has the same stance as AC toward parents. MB uses the same sentence structure: imperative tense and formal you. AC ratifies MB's utterance by repeating it and inserting it in her own narrative using “*ou*/or” which makes MB's proposition a part of AC's story, but not what she first thought. AC repeats it again twice, which suggests that this turn was truly affiliative: the stance displayed by MB is compatible with AC's own stance and with the content of the narrative.

Ex4_Fr:


  *AC_617 nous on fait notre boulot on fait
         ce qu'on peut mais
  AC_618 neuf cent élèves et si vous avez
         des problèmes vous allez voir
  AC_619 faut aller re- gueuler au rectorat
         vous avez raison allez gueuler au
         rectorat
**→ *MB_461d faites des courriers***
  AC_620 ou faites des courriers faites des
         + faut faire des courriers
         faut faire des courriers madame*


Ex4_En:


  AC_617 we do our work we do what we can
         but
  AC_618 nine hundred students if you have
         any problems you'll see
  AC_619 have to complain to the Board of
         Education you're right go complain
         to the Board of Education
**→ MB_461d send letters**
  AC_620 or send letters send + have to
         send letters have to send letters
         ma'am


#### Dislocative completion

The next example (Ex5), illustrates that ERS can also function as a completion elicited by the teller. Speaker AB is telling a story about herself and her friends who went into a nuns' dormitory when she was young. In turn 203, she reports the speech of a friend of hers. Interestingly, she does not complete the “sentence” she was reporting; the incomplete syntactic structure projects a potential continuation. But the following pattern—a filled pause, a silent pause, CM's acknowledgement signal, and a final, long silent pause before the next turn (338)—reveals a *TRP* managed by AB: AB has finished talking about the nuns and expresses a wish to continue on another topic (the events themselves). In this TRP, CM does not take the turn. After the long delay, both participants can legitimately take the floor and thus, speak at the same time. CM then produces an ERS (line 338), reporting the end of the reported speech initiated by AB. This is a typical case of completion of a dislocated structure: taken together the parts, uttered, respectively, by the storyteller and the listener, form a coherent whole, as we can see by the anaphora—the pronoun “*les*/them” corresponds to the antecedent “*ces bonnes soeurs*/these nuns”—and the verb tense (simple present).

The information added by CM is not contradictory with what AB thought, so that AB repeats it and integrates it in her own discourse, which proves that the response produced by CM is affiliative.

Ex5_Fr:


  *AB_203 et puis on avait dit §on va faire
         un truc§ etc elle avait dit oui
  CM_336 ah ouais
  AB_203 boui euh faites un truc de
         toute façon ces bonnes sœurs euh (0.305)
  CM_337 ouais (0.523)
**→ *CM_338 faut les bouger @***
  AB_204 et patin et couffin etc faut les
         bouger etc puis
  CM_339 mh
  AB_204 bfaçon tout le monde va
         croire que ce sont les
         carabins parce qu'à chaque fois
         qu'il y a des conneries qui sont
         faites
  CM_340 @
  CM_341 ouais + ouais ouais
  AB_205 c'est la faute des carabins etc
  CM_342 ouais*


Ex5_En:


  AB_203 and then we said § lets go do
         something etc she'd said yes
  CM_336 oh yeah
  AB_203 byes uh do something anyway these
         nuns uh (0.305)
  CM_337 yeah (0.523)
**→ CM_338 have to churn them up @**
  AB_204 and all that stuff have to churn
         them up etc and
  CM_339 mh
  AB_204 banyway everybody will think
         they're med students because every
         time there's trouble
  CM_340 @
  CM_341 yeah + yeah yeah
  AB_205 it's the med students' fault etc
  CM_342 yeah


While carrying out the specific action of completing the turn, CM displays her alignment with the current activity: she supports AB's narration. She provides a type of expected behavior, even *elicited* behavior. Moreover, CM expresses her stance about the story by laughing loudly just after her turn. AB's reaction gives cues about the action accomplished by CM's turn. The turn is made clear by AB's strong ratification: she repeats CM's remark, showing that it was aligned. The sequence also shows affiliation: CM's stance toward the nuns is the same as the one AB alludes to earlier (nuns are too quiet). By repeating the ERS, AB integrates it into her own story, as in the previous one.

*In sum*, Ex4 and 5 illustrate cases of strong convergence, even though they occur over a very short time span. These are cases of what we call “local” convergence. Immediately after the occurrence of the ERS, the storyteller goes back to the asymmetrical activity of storytelling by integrating the proposed reported speech into her current discourse.

#### Enumerative completion

Numerous cases of ERS can occur in an enumerative structure. Following Jefferson's (1990) analysis of lists, Selting (2004, 2007) describes enumerative as “a larger three-component structure that the list is the middle part of (…). The three components are: i/ a projection component (i.e., the formulation of a general point that projects more-to-come), ii/ a list of items, and iii/ a closure of the structure projected by i/ and ii/. The author treats the enumerative structure as a holistic entity or a “gestalt” that can be produced by a single speaker but also jointly by participants” (Selting, 2004: 212).

Reported speech can be used as an item in the enumerative structure. Similarly, ERS can be produced as an item added to the list, generally with the same voice as the storyteller's last occurrence of reported speech.

**Figure 3:**
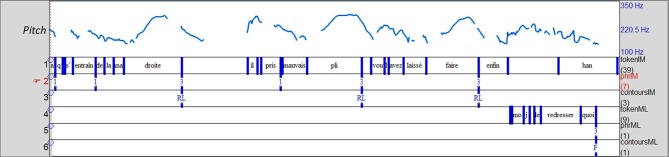
**Prosodic complementation in the enumerative structure co-produced by the storyteller (tier1) and the listener (tier4)**. The last three items -“*droite*/right,” “*pli*/habit,” and “*faire*/do”- end in an intonative unit (coded 3 on tier2) and are associated with a rising list contour, RL (tier3). The final item produced by the listener (tier4) ends in an intonative unit (coded 3 on tier5) and is associated with a final falling contour, F (tier6).

Example 6 (Figure 3) exhibits such an enumerative structure containing reported speech. IM is telling a story about the attitude of her son's teacher toward his left-handedness. She produces an occurrence of reported speech (line 727), reporting her own voice, and then creates a reported dialogue, reporting the teacher's speech. Figure 3 illustrates that turns 728–729 are composed of a list of several items (3 in 728 and 2 in 729) each corresponding to a prosodic unit associated with a typical rising list contour (RL or L+H*H% in *auto-segmental metrical* terms) (Portes et al., 2007). The occurrence of ERS (line 691) appears as the third component of the enumerative structure (closure) initiated by “*et*/and” and closed by a discourse marker “*quoi*/you know” spoken with a falling contour (F or L%). The listener thus, ended the enumerative structure initiated by the storyteller. This structure was co-elaborated by the participants both discursively and prosodically (as illustrated by Figure 3). This case of *prosodic complementation* is a form of *prosodic orientation* in which the last falling contour is expected, after the rising contours, to close the enumerative structure (Szczepek Reed, 2006: 61). As a result of the discursive and prosodic orientation, the response is aligned.

In addition, ML's stance toward the events and toward the teacher (critical) is the same as the narrator's, and her response is thus, also affiliative. Like reformulation cases, the storyteller does not always ratify the added affiliative item. The lack of ratification here is equivalent to a basic acceptance, affiliation being the response preferred and expected by the storyteller.

Ex6_Fr (Figure 3):


  *IM_726 @je cours à l'école je lui dis
  IM_727 vous savez que ça fait cinquante
         ans qu'on laisse les enfants écrire
         avec la main qu'ils veulent hein
  ML_690 @
  IM_728 oui oui mais vous comprenez il
         aura une écriture horrible je ne
         peux pas tolérer euh une chose
         pareille euh il faut absolument
         qu'il s'entraîne de la main droite
         euh
  IM_729 il a pris un mauvais pli euh vous
         l'avez laissé faire enfin
**→ *ML_691 et moi je vais le redresser quoi***
  IM_730 han
  IM_731 oh là là je dis bon ben écoutez
         dans ce cas nous n'avons plus @
         rien à nous dire @ je le change
         d'école immédiatement quoi je l'ai
         encore changé d'école*


Ex6_En (Figure 3):


  IM_726 @I'm rushing to school + I tell her
  IM_727 now you know it's been fifty years
         since children have been allowed to
         write with whatever hand they want
  ML_690 @
  IM_728 yes yes but you understand he'll
         have terrible handwriting I
         can't tolerate uh such a thing he
         absolutely has to practice with the
         right hand uh
  IM_729 he got into a bad habit uh you let
         him do it actually
**→ ML_691 and I'm going to fix it you know**
  IM_730 uhn
  IM_731 oh I say good well listen in that
         case we don't have anything more
         @ to talk about @ I'll switch
         his school immediately you know
         I switched his school


**Figure 4:**
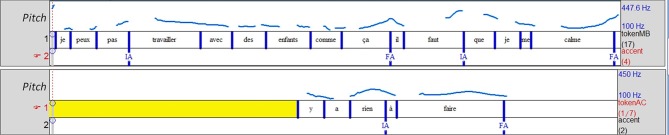
Prosodic matching of the *accentual arch* (bounded by an initial accent IA or LHi and a final accent FA or H*/L* on part of the utterance). By using the same scale (100–550 Hz for the different speakers) we can see that the storyteller (top two lines) is producing the same accentual configuration with an expanded span (second occurrence) while the listener (bottom line) is producing the same one but in a very compressed span. The reiteration (by the storyteller) of the same configuration and the prosodic matching (by the listener) both contribute creating a list effect.

Example 7 (Figure 4) presents another enumerative structure, but with different consequences for the sequence's degree of convergence. MB is telling a story in which she reports her own speech. The structure is composed of three items, and AC proposes an additional item with a similar prosodic configuration, as shown by Figure 4. Here, the list effect is created by the reiteration of a typical global configuration in the intonative units from the storyteller. This configuration is characterized by an initial and a final accent that form an *accentual arch* (Fonagy, 1979; Di Cristo, 1999) that functions as a cohesive mark (Figure 4). The copy of this configuration in the ERS illustrates a case of *prosodic matching* (Szczepek Reed, 2006). Consequently, the turn is clearly oriented toward MB's previous talk, showing alignment both to the storytelling and the device: a continuation of the enumerative structure and the same (reported) voice in a similar accentual configuration as MB.

AC attempts to display affiliation by reporting MB's speech: she expresses a stance corresponding to MB's, through her use of MB's voice. Despite the opportunity for affiliation, this element not only does not receive an explicit verbal ratification, it is ignored. This can be explained by the fact that it functions as feedback: although it corresponds to prosodic matching on the prosodic figure exhibited (*accentual arch*), we can see that this configuration not only overlaps with MB's turn but is also in a very compressed pitch range. In contrast, MB's three arches are produced in an increasingly expanded pitch range that seems to function as a turn-holding cue. Moreover, MB's next turn is oriented toward her own previous speech, and not toward AC's turn since 417 is a completion of 416 and thus, cannot complete ERS (475). As a result, the sequence appears as a non-affiliative sequence, despite AC's attempt to produce an expected stance.

Ex7_Fr (Figure 4):


  *MB_415* je lui ai téléphoné à la directrice
         disant tu as absolument raison je
         me suis rendue compte que j'étais
         pas dans mon état normal
  *AC_474* ah ouais
  *MB_416* je peux pas travailler avec des enfants comme ça il faut que je me calme
**→ *AC_475* y a rien à faire**
  *MB_417* pa(r)ce que sinon je peux pas @


Ex7_En (Figure 4):


  MB_415 I called the principal saying
         you're absolutely right I realized
         I wasn't in my normal state
  AC_474 oh yeah
  MB_416 I can't work with children that way
         I need to calm down
**→ AC_475 nothing works**
  MB_417 because if not I can't @


In sum, Ex5, 6 and 7 show ERS that is strongly oriented toward the previous turn: the ERS utterances are aligned in supporting the storytelling activity in progress and in completing the discursive devices used (dislocated and enumerative structures). For enumerative structures, the degree of convergence depends on the storyteller's possible orientation on the added item.

#### Relying on introductory formula

Our data present other cases illustrated by Ex8 and 9. When the storyteller produces an introductory formula for his/her reported speech, the listener can then rely on that formula or on the discourse particle that begins the reported speech, to produce reported speech using the same voice as the introductory formula.

In the example below (Ex8), AB is telling a story about running a red light, and she is about to report the thoughts that she and her friends had at one point in the story. She produces an introductory formula, “*on se dit/*we think,” and then the discourse marker “*bon*/well”; CM then produces an ERS (line 366): it is aligned (adapted to the narrative), and it is also adapted to the device. It fits perfectly with the storyteller's introduction, and it reports the supposed thoughts of AB and her friends. Not only is the element not ratified but especially AB orients toward her own idea, and consequently creates a repair sequence by repeating her own introductive formula. Therefore, despite CM's attempt to match AB's stance, her response is not affiliative.

Ex8_Fr:


  *AB_223 et on dit § on brûle le feu rouge et
         puis je sais plus moi ou quelqu'un
         a dit oh c'est pas la peine écoute
  CM_364 mh
  AB_225 puis à un moment donné on regarde
         derrière nous y avait une bagnole
         de flics
  CM_365 ah d'accord
  AB_226 et là on se dit
**→ *CM_366 ah @ depuis quand ils sont @ là
         déjà mh mh @***
  AB_227 bon
  AB_228 @
  AB_230 on s'est dit déjà une heureusement
         qu'on n'a pas brûlé le feu rouge*


Ex8_En:


  AB_223 and we say let's run the red light
         and then I forget or someone said
         hey it's not worth it look
  CM_364 mh
  AB_225 then at one point we look behind us
         and there was a cop's car
  CM_365 oh okay
  AB_226 and then we think
**→ CM_366 oh @ + how long have they @ been
         there mh mh @**
  AB_227 well
  AB_228 @
  AB_230 we thought first luckily we didn't
         run the red light


**Figure 5:**
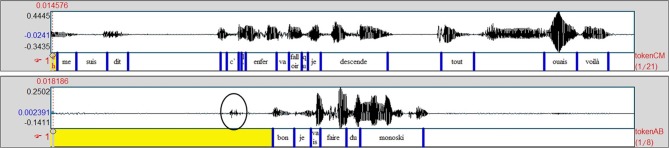
**Simultaneous reported speech from the storyteller and the listener**.

The next example (Ex9, Figure 5) shows a very similar case in terms of form. Here, CM is telling a story about how her ski broke when she was skiing. At this point in the story, CM is about to produce reported speech about her own thoughts. She produces an introductory formula, and the discourse marker “*mais*/but,” which AB immediately follows with ERS using the same voice to show that she is reporting CM's thoughts. After a long pause of 700 ms, which is visible on Figure 5, both participants simultaneously attempt to complete the thoughts initiated by the introduction of the reported speech: CM utters “*mais*/but” just as AB produces a click (relevant cue for taking the floor, circled in Figure 5).

The difference from the previous example is evident in the listener's change of tonality (i.e., the proposal of a “mood for the joke,” Norrick, 2000: 174). This is a case of completion used to make fun of someone. While apparently reporting CM's thoughts, AB takes some distance from the normal strand of the story, and she displays a faint affiliation. The mocking tonality of the utterance apparently makes the sequence disaffiliative (AB knows that CM did not break her ski on purpose). In fact, the humorous dimension of the ERS allows her to make fun but also to make evident the absurdness of the situation described by CM. So in doing this, AB tells CM that her story was actually an unusual event, and consequently, CM actually correctly achieves the instructions given for the task, and AB has the same opinion as CM concerning the events: it is an unusual/absurd story. In the following turn the speaker ratifies the mockery (“*ouais voilà*/yeah there”), then goes back to the normal strand of the narrative. The reversal of roles that contributes to defining convergence in this paper is clearly illustrated by this example: AB takes the turn and thereby becomes the temporary main speaker. This change is also obvious at the gestural level (see Figure 1) where the behaviors are reversed: in Figure 1C, AB (left) looks away and produces a large illustrative gesture during her ERS, whereas CM (right) directs her gaze at AB and stops moving. This results in a local convergence sequence.

Ex9_Fr (Figures 1, 5):


  *CM_463 et en fait euh le problème c'est
         que si tu peux plus du coup je
         pouvais plus chausser j'étais tout
         en haut de la station je pouvais
         plus chausser me suis dis mé-
         (0.700)
  CM_465 mais c'est l'enfer
         va falloir que je descende
→ ***AB_294 CLIC bon je vais faire du monoski***
  CM_466 tout euh ouais voilà
  CM_467 ou euh donc en fait j'ai déchaussé*


Ex9_En (Figures 1, 5):


  CM_463 and in fact uh the problem is that
         if you can't do it anymore so I
         couldn't put them on anymore I was
         way above the station I couldn't
         put them on anymore I thought mé-
         (0.700)
  CM_465 but this is hell I'll have to go
         down
**→ AB_294 CLICK right well I'm going monoskiing**
  CM_466 all uh yeah there
  CM_467 or uh so in fact I took them off


#### Reporting other voices

In the next section, the listener produces ERS which reports other voices. This creates a reported dialogue between the characters in the story: the listener transforms an isolated occurrence of reported speech into a reported dialogue involving different voices.

In the next example (Ex10), CM is telling a story from her childhood, when she got confused between a dummy and a real employee in a store. She reports that the lady said “*bonjour*/hello,” which was very surprising to her, because she thought that it was a plastic mannequin. She gives many cues of her own stance toward the events, speaking for instance of a “*moment de peur*/scary moment.” This ERS constitutes an aligned and affiliative response to the story. Moreover, in this sequence, the participants are both strongly involved in the activity (high intensity of speech, loud laughing). It appears as a convergent moment in the interaction.

Ex10_Fr:


  *CM_856 j ça était vraiment un un moment de
         peur
  CM_857 immense quoi tu vois ne pas sentir
         heu
  CM_858 un mannequin habituel tu vois mais
         de sentir une vraie main dans ma
         main
  CM_859 et la la nana était très sympa elle
         m'a regardé elle m'a dit bonjour
         mademoiselle putain ah @ tu sais la
         la
  AB_618 mh*
→ ***AB_619 @ le mannequin parle***
  *CM_618 bpanique je l'ai plus jamais
         recommencé après la panique
  CM_620 @
  AB_621 @*


Ex10_En:


  CM_856 I it was really a scary moment
  CM_857 huge you know not to feel uh
  CM_858 a normal dummy you see but to feel
         a real hand in my hand
  CM_859 and the the lady was very nice she
         looked at me she said hello miss
         fuck oh @ you know the the
  AB_618 mh
→ **AB_619 @ the dummy talks**
  CM_618 bpanic I never did that again the
         panic
  CM_620 @
  AB_621 @


Making complaints or telling amusing stories makes it possible for the participants to employ a humorous tonality (Bertrand and Priego-Valverde, 2011). Some stories are told entirely in a humorous way. We now examine an example of this kind: the setup is similar as in the previous example, but here it occurs in a humorous sequence.

Example 11 presents an instance of humorous reported speech that appears in a humorous story. The listener adopts the same tonality, so the response is aligned with the activity but also adapted to the tonality. LJ is telling a story about some strange men he met who he thought were going to kill him. This is a cue for his stance about the characters and the story. AP then produces ERS which “reports” the men's offer. To do this, AP uses the same word as LJ used in the penultimate turn, “*prospection-prospecter*/exploring.” This is a way of showing his orientation toward the previous sequence. The verbal content and the stance AP displays are the same as the one that LJ expressed in the previous turn (430): “*ils vont me tuer*/they're going to kill me,” but AP has used a new voice to reflect LJ's stance.

The participants previously built a humorous sequence, characterizing the enunciator as a “*voileur du bois*/rapist of the woods.” In their study about prosody and humor, Bertrand and Priego-Valverde (2011) established, for this same excerpt, that the ERS uses a stereotyped voice linked to the characterization. The humorous dimension of the excerpt causes overbidding, in which the stance is “exaggerated.” It still constitutes a highly affiliative response, and the laughter produced by both speakers are a cue to this interpretation. The humorous dimension of the listener's production is recognized by the storyteller, who ratifies it. Then the participants go back to the main activity in which they were engaged: LJ's storytelling. We thus, consider this to be a convergent sequence.

Ex11_Fr:


  *LJ_429 voilà qu'ils me proposent de
         d'aller euh avec eux en prospection
         dans je sais plus où euh
  LJ_430 dans l'Esterel ou je sais pas euh
         pendant euh le m @@ pendant le mois
         d'août tu sais je me suis dit mais
         attends hé ils vont me tuer quoi@@
  AP_498 a @*
→ ***AP_499 viens prospecter avec nous petit @
         hé hé hé***
  *AP_499 a @*


Ex11_En:


  LJ_429 there they offered to to take me
         uh with them exploring in I don't
         remember where uh
  LJ_430 on the Esterel or I don't know uh
         during uh the m @@ during the month
         of August you know I thought but
         hey wait they're going to kill me
         you know @@
  AP_498 a @
→ **AP_499 come exploring with us little one @
         ha ha ha**
  AP_499 a @


Example 11 appears in a sequence already established as humorous. In the next cases, ERS appears after reported speech and is used as a way of introducing a humorous tonality into a sequence which had a “neutral” tonality up to that point. We examine these examples in a separate subsection because the relevant information is not primarily the voice used, but the fact that the listener distances him/herself from the story and produces a response that introduces an “enunciative source” other than his/her own voice.

#### Introducing a new tonality

Example 12, AB is telling a story in which a friend of hers fell down. She reports what the friend said in a reported dialogue and then what she and her friends answered. After giving specific evaluative responses, CM produces ERS—“*tu peux crever*/you can drop dead”—that overbids “*on se casse*/we're outta here.” Despite its similarity to Example 5 (same speaker in the same narrative), this ERS is not ratified by the storyteller. This may be due to the fact that CM's language was too strong; she authorizes this language by using a humorous tonality, but AB does not judge it to be acceptable or include the response in her story. Therefore, this is not an affiliative response.

Ex12_Fr:


  *AB_214 il était à moitié allongé par terre
         avec sa jambe comme ça en disant
         oh j'ai mal j'ai mal j'ai mal + on
         a dit on s'en fout on se barre et
         tout alors il a quand même réussi
  CM_354 @ oh putain excellent @*
→ ***CM_356 tu peux crever***
  *AB_217 il a quand même réussi à nous
         suivre*


Ex12_En:


  AB_214 he was halfway stretched out on
         the ground with his leg like that
         saying oh it hurts it hurts + we
         said we don't care we're outta here
         and all well he still managed
  CM_354 @ oh shit great @
→ **CM_356 you can drop dead**
  AB_217 he still managed to follow us


As often acknowledged, even in a very cooperative interaction such as a conversation, instances of competition or disagreement can appear. Until this point in the analysis, we have considered sections in which participants agreed. However, ERS can also appear in sequences of disagreement.

In the following example (Ex13), LJ is telling a story about archeological digs, which AP negatively evaluates by summarizing the story in an absurd way. From this point on, then they disagree. LJ tries to explain the reasons why the dig is not a scam. He uses reported speech to describe the content of a “*diplôme/*diploma” given to participants. AP then produces two occurrences of ERS (421, 422), simulating the content of the diploma and reformulating what LJ has just said. Each of AP's reported speech occurrences takes a humorous, mocking tone. Not only is there no affiliation in this case, since they do not express the same stance, but there is disaffiliation between participants since their stances are radically opposed. As we can see here, although ERS is generally used by participants in order to (attempt to) affiliate, it can also be used in an oblique way to show disaffiliation.

Ex13_Fr:


  AP_418 tu payes pour fouiller
  LJ_341 tout ça
  LJ_342 ouais tu tu tu payes ouais mais a
         tu sors t'as une espèce de pas un
         diplôme je sais pas mais enfin d c'
         c'est j'ai fouillé à machin et
  AP_419 @ tu payes pour faire le manoeuvre
  AP_420 ^*^ super
  LJ_344 et là bon c'est c'est
→ ***AP_421 @ j'ai un diplôme de fouilleur***
  *LJ_345 si tu veux c'est c'est fait euh*
→ ***AP_422 @ j'ai tenu une pioche pendant une
         semaine***
  *LJ_346 c'est un chantier école
         c'est-à-dire que t'as des cours
         t'as des cours sur la céramique euh
  AP_423 ah hum hum hum
  AP_424 mh mh ah ouais OK ouais*


Ex13_En:


  AP_418 you pay for excavations
  LJ_341 all that
  LJ_342 yeah you you you pay yeah but a you
         go out you have some kind of not a
         diploma I dunno but d it it's I dug
         with a whatchamacallit
  AP_419 @ you pay to have the manœuvre done
  AP_420 ^*^ great
  LJ_344 and there well it's it's
**→ AP_421 @ I have an excavator**'**s diploma**
  LJ_345 if you want it's it's done uh
**→ AP_422 @ I held a pickaxe for a week**
  LJ_346 it's a school construction site
         meaning that you have some courses
         you have some pottery courses uh
  AP_423 ah huh huh huh
  AP_424 mh mh oh yeah OK yeah


In most cases, ERS appears in the close environment of reported speech from the storyteller. It frequently follows (or is produced simultaneously with) the storyteller's reported speech, but occasionally it can anticipate it.

#### Anticipating a reported speech utterance

In Example 14, the storyteller ML is not currently producing reported speech. She describes an attitude of the characters in her story by using “*genre/*a sort of” (675). More than an introductory formula, “*genre*” carries a type of representation of the “animated character” (Couper-Kuhlen, 1999) that can infer a type of stance (“*dégoûtés*/disgusted”). IM repeats “*genre*,” and relies on it to reformulate the adjective “*dégouté*” into direct reported speech, corresponding to the same critical stance toward the attitude described by ML. Even though ML is not reporting speech at this point, there are still similarities in the content and structure (“genre”). As reported speech from the storyteller elicits ERS in other cases, “*genre*” (produced by the storyteller) here elicits ERS, which ML ratifies with “*ouais*/yeah.” Although ML does not produce reported speech before the ERS, she does so in the following utterance. In this case, ERS could encourage the storyteller to use reported speech. This is a local convergent sequence.

Ex14_Fr:


  *ML_673 et alors j'arrivais à neuf heures
         moins le quart dans la salle
         commune ils sont tous euh + assis
  ML_674 comme ça
  ML_675 genre euh + dégoûtés quoi*
→ ***IM_697 genre va falloir attaquer la
         journée***
  *ML_676 ouais et alors à neuf heures cinq y
         en a une qui dit on va sonner non*


Ex14_En:


  ML_673 and so I was walking into the
         common room at eight forty-five
         they're all uh sitting there
  ML_674 like that
  ML_675 a sort of uh + disgusted you know
**→ IM_697 a sort of we've got to attack the
         day**
  ML_676 yeah and then at nine-o-five one of
         them said time to ring no


#### Creating an oblique sequence

In this subsection, ERS is used to initiate a new *oblique* sequence (Stivers, 2008). We show that it results in temporary disalignment, contrary to the previous examples.

In Example 15, LJ is telling a story in a neutral tone. AP produces an ERS utterance (line 844) using the voice of a fictitious psychoanalyst asking a question. This remark is presented as humorous and as non-aligned, considering the absurd scenario it brings to life. AP then continues to use a humorous tone, producing a second occurrence of ERS (line 845): an answer to the question, given by a fictitious patient. Then LJ continues and overbids on this topic, producing another question whose enunciator is still the fictitious psychoanalyst. At this point, he agrees to engage in this new activity of *joint fantasy* (Kotthoff, 2006), together with the listener, so that they re-align toward the new activity. By doing this, LJ orients more explicitly to the sexual dimension, while he refers to dialogues of a famous movie (“Airplane!”). AP then produces a new reported speech utterance, still from the same movie. Since the participants imagine the same situation together—a situation which has digressed from the normal frame of the story and which includes shared knowledge—they show affiliation. After this oblique sequence, they go back to the initial activity of storytelling.

The humorous sequence is constructed from the listener's initiation of an oblique sequence, which causes disalignment from the current activity. The storyteller's realignment with this new activity leads to their co-elaboration of a highly affiliative sequence resulting in a highly convergent sequence overall.

Ex15_Fr:


  *LJ_778 fait un truc tu sais un vague contour
         quoi et ça ressemblait à un oiseau
         photocopié quinze mille fois un peu
         colorié
  LJ_779 tu dis mais attends un gamin il fait
         ça déjà tu lui files deux baffes quoi
  ***AP_844 qu'est-ce que ça t'évoque***
  ***AP_845 eh ben là disons que euh je pense
         plutôt à ma mère euh***
  LJ_780 et euh et
  ***AP_846 @ et à mon attirance euh***
  ***LJ_781 et en plus euh tu aimes les films de
         gladiateurs @***
  ***AP_847 oui as-tu déjà été dans les bains
         turcs euh****


Ex15_En:


  LJ_778 do something ya know a rough outline
         like that looked like a bird
         photocopied fifteen thousand times
         a little colored in
  LJ_779 you say but wait if a kid does that
         you know you'd slap him twice like
  **AP_844 *what does that remind you of*
  AP_845 *well there let's say that uh I think*
  *of my mother uh***
  LJ_780 and uh and
  **AP_846 *@ and of my attraction uh*
  LJ_781 *and plus uh you like gladiator movies*
  AP_847 *yes have you already been to a Turkish
         bath uh***


In Example 16, SR is reporting his experience when he lived abroad: he expected to be treated as a foreign agent (to be reimbursed more quickly, because foreign agents are not registered in the computer files). But he was treated as a French agent. In turn 680, EB produces a specific response “*et donc tu étais français*/so you were French,” which can be considered as a completion, oriented toward SR's previous turn. SR then reports his own reaction to the office's statement: “*non*/no” in turn 563. At this point, the participants begin to laugh and continue doing so until the end of the sequence. EB then completes the interjection reported by SR with ERS, while developing the idea: “*c'est un autre*/that's somebody else.” Reporting what SR could have said in this situation, EB displays his understanding of the situation and his affiliation. SR then produces “*je suis de Glasgow*/I'm from Glasgow,” which does not orient toward EB's turn but is a completion of his own previous turn. EB then continues the idea SR has just introduced: “Simon Rivière” is an ERS utterance produced with a phonetic modification, a cue to code-switching. SR then also produces reported speech in which he uses the same accent and the English words “from Glasgow University,” consequently orienting toward EB's turn. EB then repeats “from Glasgow,” again showing alignment. In turn 566, SR produces reported speech which is then followed by EB's very similar turn, which he begins with the same structure, “that's not the.”

This alignment and affiliation results in a highly convergent sequence in which ERS is associated with other devices, such as lexical similarity (other-repetition), language similarity (code-switching), syntactic similarity, and much loud and long laughter (@). These various devices display affiliation (see Bertrand and Priego-Valverde, 2011 for prosodic matching). This sequence is co-elaborated to such a degree that the participant's roles are confused. In contrast to the asymmetry of the previous story, the oblique sequence here exhibits symmetry of roles in which either participant could be the main speaker.

Ex16_Fr:


  *SR_559 donc quand j'étais à Glasgow et
         j'étais hyper content parce que je
         me suis dit ah pour une fois
  SR_560 je vais être euh remboursé euh
         instantanément
  SR_561 et euh et en fait j'ai pas eu de
         chance ils avaient gardé euh mon nom
         sur l'ordinateur
  EB_680 et donc tu étais français
  SR_562 et j'étais euh donc euh ils ont dit ah
         non mais lui on a un dossier c'est bon
  EB_680 a @
  SR_563 alors j'avais dit non @
  **EB_681 @c'est un autre
  SR_564 je suis de Glasgow @
  EB_681 a @
  EB_682 Simon Rivière @
  SR_565 @ from Glasgow University @
  EB_683 @ from Glasgow @
  EB_684 you remember me
  EB_684 a @
  SR_566 @ that's not me @ the French one no no
         no @
  EB_685 @ not euh that's not the same guy
  EB_686 @ I have heard of this guy**
  SR_566 a @
  EB_686 a @
  SR_567 ouais*


Ex16_En:


  SR_559 so when I was in Glasgow and I was
         ultra happy because I thought oh for
         once
  SR_560 I'm going to be uh paid uh instantly
  SR_561 and uh and in fact I didn't have a
         chance they had kept uh my name in the
         computer
  EB_680 and so you were French
  SR_562 and I was uh so uh they said ah no but
         we have his file it's ok
  EB_680 a @
  SR_563 so I said no @
  **EB_681 @ that's somebody else
  SR_564 I'm from Glasgow @
  EB_681 a @
  EB_682 Simon Rivière @
  SR_565 @ from Glasgow University @
  EB_683 @ from Glasgow @
  EB_684 you remember me
  EB_684 a @
  SR_566 @ that's not me @ the French one no no
         no @
  EB_685 @ not uh that's not the same guy
  EB_686 @ I have heard of this guy**
  SR_566 a @
  EB_686 a @
  SR_567 yeah


Lastly, in some cases, the listener repeats reported speech produced by the storyteller. These other-repetitions can take similar forms as ERS.

Example 17 presents a story told by AB. She reports a reported exchange between herself and a friend of hers. CM repeats what AB has just said. However, this is not a case of ERS, even if the use of deixis is consistent with the story being told, and not with the situation in which the participants are being recorded. Since AB has just uttered exactly the same sentence, the second turn is a repetition, with a “savoring” evaluative function (Tannen, 1989, 2007). The listener does not provide a stance toward the events but rather an evaluation of AB's words themselves. Consequently, we cannot infer any consequences about affiliation.

Despite their similar forms, other-repetition is different from ERS in the sense that it appears in second position: the element that is repeated has already been produced by the main speaker, and it is not invented by the listener thanks to shared knowledge.

Ex17_Fr:


  *AB_5 65 et je trouvais ça super joli et
         je me rappelle je devais être avec
         Annie cette fois-ci elle m'avait
         dit ah mais c'est horrible et tout
  CM_789 ah ouais c'est ouais c'est sympa
         ouais
  CM_790 ouais ah ouais c'est sympa
  AB_566 qu'est-ce tu vas faire de ça j'ai
         dit ah mais euh
  CM_793 des jambes @*
→ ***AB_567 ça me plaît assez*
→ *CM_794 @ ça me plaît assez***
  *AB_568 @
  AB_569 @ et en fait @ dans la maison où
         j'habitais à La Rochelle*


Ex17_En:


  AB_565 and I thought it was super pretty
         and I remember I must have been
         with Annie at the time she had told
         me oh but it's horrible and all
  CM_789 oh yeah it's yeah it's nice yeah
  CM_790 yeah oh yeah it's nice
  AB_566 what are you going to do with it I
         said oh but uh
  CM_793 some legs @
→ **AB_567 I like that well enough
→ CM_794 @ I like that well enough**
  AB_568 @
  AB_569 @ and in fact @ in the house where
         I lived in La Rochelle


## Discussion

In this paper we examined how participants in conversation build convergent sequences in accordance with the concepts of alignment and affiliation (Stivers, 2008). We highlighted a type of specific response (Bavelas et al., 2000) expressed by the listener in storytelling. This specific response is a kind of reported speech that occurs during a narrative and that we term “Echo Reported Speech” (ERS), insofar as it is produced as an “echo” to the ongoing narrative. Usually, reported speech is a device used by the storyteller to report not only words but also thoughts (Klewitz and Couper-Kuhlen, 1999) to achieve certain goals during talk-in-interaction. We showed here that when the storyteller had given enough information, the listener could produce ERS him/herself by which she/he could display (dis)alignment and (dis)affiliation in orienting respectively, to the current activity and the expected stance. Moreover, the use of reported speech by the listener represents a change of discursive role that is also of crucial importance to the study of convergence: reporting speech consists of producing words that have been said in another situation. Since the listener cannot have heard these words before, his/her reported speech (invented) provides evidence that he/she is temporarily taking the place of the main speaker.

Following Stivers (2008) alignment pertains to the activity being carried out by the participants of an interaction. Affiliation concerns the stance of participants: a response that displays the same stance as that of the other participant is an affiliative response. ERS signals alignment: it constitutes an adapted response, presented as specific to the ongoing narrative. In many cases, the storyteller him/herself elicits this type of response. Holt (2000) argues that because of its double function, direct reported speech allows the “reporter” to appear to accurately report the speech of another speaker, while simultaneously commenting upon the reported utterance. In doing so, he/she implicitly displays his/her own stance and elicits an explicit display of that stance by the listener. Following Stivers (2008), displaying the same stance as the other participant is an affiliative response. Listeners producing ERS thus, display their stance toward the story being told.

In other cases, ERS is used for alignment and affiliation when the listener displays a stance similar to the storyteller's. These sequences are potential places for the emergence of convergent sequences. Alignment without affiliation occurs in cases where the main speaker does not react to the ERS in the following turn, but continues his/her own narration with no explicit sign (verbal, laughter) of an orientation toward the listener's production.

Finally, disalignment linked to a change of activity (as in a humor sequence) can be associated with affiliation and lead to highly convergent oblique sequences, such as Joint Fantasy. Alternatively, this type of sequence can fail to lead to affiliation, in which case it results in a lack of convergence.

In this study of convergence phenomena, we did not discuss the concept of similarity between one participant's production and that of the other participant. Similarity and convergence are often considered synonymous. We argue from an interactional point of view that while the various manifestations of similarity can point to convergence, convergent sequences take on a much greater variety of forms, due to the different activities deployed and the participant's differing roles, among other things (see Bertrand et al., 2013, to show that “gestural” similarity is not sufficient to describe convergence). However, ERS itself can be seen as a type of similarity, in the sense that it co-occurs with Reported Speech in such a way that both participants use the same discursive device. Moreover, similarity can also be observed through the use of voices: the listener uses the same voice as the one animated by the storyteller in his/her own story.

Tannen (1989, 2007) argues that “when a listener utters a line of dialogue for a story she isn't telling, that dialogue certainly cannot be considered reported.” But if we take the delivery of ERS into account (change of verb tense, matching use of personal, spatial, and temporal deictics relevant to the narrative rather than the situation) into account, ERS appears as a type of reported speech. Moreover, from an interactional point of view, producing reported speech is a way of representing an encounter between individuals. In ERS, the listener represents an encounter he/she has not witnessed. But ERS is an invention, and as in every type of invented reported speech, the encounter described is a fictitiously anchored one. ERS consists of reporting the speech that a character could have said in a precisely defined situation. The listener has understood the story told so well that he/she is totally aligned and becomes able to produce speech that a character of the story could have said. This invented reported speech is however, consistent with Vincent and Dubois' definition (1997) of invention reported speech even though these authors only consider the storyteller's point of view. Ultimately, whether the status of ERS is reported speech or not, it would still appear as an aligned and/or affiliative response, and it would have the same consequences on the sequence's degree of convergence.

More generally, in highly convergent sequences, ERS is often associated with many other similarity based devices (other-repetition, prosodic matching, simultaneous laughter, etc.). This highlights the complexity of the convergence phenomena that the notions of *alignment* and *affiliation* enable us to capture. Such an interactional orientation framework provides insight into interactional convergence. Moreover, a sequential analysis supplies some cues for gaining access to expressed opinions and thoughts, which are studied using cognitive models. Among these models, Pickering and Garrod (2004) proposed a psychological model of interactional alignment in which the alignment of mental representations has consequences on alignment at several linguistic levels. The alignment of representations is a requirement for successful interaction. In a conversation, for example, if the participants have aligned their representations, their linguistic utterances will be aligned too (phonetically, syntactically, and semantically). In any case, participants who try to align may fail, in which case they can use repair sequences to establish alignment. Finally if they still do not manage to align their representations, they begin to explicitly talk about the misalignment in order to resolve it. In this context, the contribution we present here provides support for this model: using the same discursive device as the main speaker is a way for the listener to align at the interactional level. When displaying affiliation, the ERS allows the listener to express his/her own stance toward the events. It consequently can be a way of expressing opinions or representations.

The relationship between affiliation and alignment is highlighted by Stivers (2008), who assumes that affiliation requires alignment. The author explains that since affiliation means that the listener knows which stance the teller has toward his/her own story and knows that a similar stance is the preferred response, the participant has received enough information to be able to produce the affiliative response, which necessarily occurs after he/she has understood the story (exhibited by the alignment). However, in some of our examples (Joint Fantasy, among others) affiliation co-occurs with disalignment. This appears to contradict Stivers' argument that alignment is required before affiliation. But in fact, in the cases we observed, the main speaker tells a story, to which the listener aligns (thanks to generic responses). At a certain point in the story, the listener produces a disaligned response, which constitutes a proposal to change the activity. The storyteller can refuse this proposal of an oblique sequence: in that case, the disalignment caused by the humorous ERS, for example, is effective and needs to be resolved by the listener him/herself, who then realigns, going back to the previous activity of listening to the story. In these cases, there is a lack of convergence. But in other cases, the storyteller accepts the oblique scenario and consequently agrees to temporarily set aside his/her narrative. Meanwhile, the storyteller (who is no longer the main speaker) aligns with the new activity proposed by the listener. We have mainly observed this kind of sequence in Joint Fantasy (Kotthoff, 2006). It is not surprising that highly convergent sequences co-occur with humor, since alignment and complicity are prerequisites of humor.

Concerning the turn-taking organization, ERS also plays a role that affects convergence. Conversation is a symmetrical interaction in which participants play symmetrical roles: the differences between speakers are minimized. No differences in their status in the interaction are created *a priori*. But in the activity of storytelling, the roles are highly asymmetrical: each participant has to accomplish different tasks, conditioned by their role as storyteller or as listener. We show here that ERS causes a change in roles: the listener produces reported speech -which is normally a device used by the main speaker-, thereby temporarily taking the main speaker's place. This role change decreases the differences between the participants and contributes to the potential emergence of convergent sequences. In the case of highly convergent sequences such as humorous ones, the participants no longer hold two asymmetrical roles at all.

Joint fantasy is the most convergent sequence in this context, which includes the task in which the participants are involved, the relationship between them, and also the cultural context. In some cultures, symmetry between participants is not conceivable. In other cultures (Danziger, 2006) fantasy may not be the preferred sequence: alignment and affiliation, together with the symmetrical status of participants, may result in another type of convergent sequence.

Finally, this study demonstrates the importance of clearly defining the notion of convergence in conversational data. This notion, apparently easy to capture through the notion of similarity, cannot be reduced to a single concept. Both alignment and affiliation are relevant notions for describing convergence phenomena. By investigating other specific responses, we are currently enriching our description of the recipient design in terms of alignment/affiliation. This leads us to improve our understanding of interactional convergence and more generally the organization of conversations.

Transcription conventions:

@: laughter
@@ … @@: laughing sequence
Underlining: overlapping speech
(0.25): duration of pause (in seconds).

### Conflict of interest statement

The authors declare that the research was conducted in the absence of any commercial or financial relationships that could be construed as a potential conflict of interest.
